# What is e-Health (2): The death of telemedicine?

**DOI:** 10.2196/jmir.3.2.e22

**Published:** 2001-06-22

**Authors:** Vincenzo Della Mea

The first time I heard the term "e-health" I was at the 7th International Congress on Telemedicine and Telecare in London, at the end of November 1999. John Mitchell from Sidney, Australia, spoke about a national government study whose main result was the recognition that "cost-effectiveness of telemedicine and telehealth improves considerably when they are part of an integrated use of telecommunications and information technology in the health sector." [1]. This led to the identification of "e-health" as an umbrella term, with definitions such as "a new term needed to describe the combined use of electronic communication and information technology in the health sector... the use in the health sector of digital data - transmitted, stored and retrieved electronically - for clinical, educational and administrative purposes, both at the local site and at distance" [2].

In this talk, e-health was introduced as the death of telemedicine, because - in the context of a broad availability of medical information systems that can interconnect and communicate - telemedicine will no longer exist as a specific field. The same could also be said for any other traditional field in medical informatics, including information systems and electronic patient records. e-health presents itself as a common name for all such technological fields.

Mitchell also pointed out that "e-health can be considered to be the health industry's equivalent of e-commerce," and this could be one key for understanding the sense of e-health: just medical informatics and telematics on the shop shelves, a fashionable name for something already existing but otherwise difficult to sell.

Without arguing anything about the consequentiality of the facts, in December 1999 the subtitle of *Telemedicine Today*- a non-peer-reviewed journal - changed from "Where healthcare + telecommunications converge" to "The eHealth Newsmagazine," and just some months later, even the *Telemedicine Journal*- a scientific, peer-reviewed journal - added an "and eHealth" to its title. Nice name? Fear of being left out of a possibly-new field? The Ace Allen editorial that introduces the change in the subtitle of *Telemedicine Today* [3] sounds slightly bitter: during the time the telemedicine market exploded, *Telemedicine Today*'s name suddenly changed, perhaps to satisfy the hundreds of healthcare-related dotcoms looking for a buzzword.

Shortly after the above changes, E. Rosen [4] rationally explained some differences related to the use of the words "telemedicine" and "e-health." Investors look for investments that can produce high returns even after several years. From this point of view, the specific term of telemedicine seems inadequate, as it identifies a market niche, while e-health, as any "e-thing", seems more open and promising (just like anything without a clear meaning). Rosen also points out the hardware-centric aspects of telemedicine, which is based on the traditional equipment sales model, while e-health is apparently oriented to service delivery, which is more interesting on the business side. The final remark of Rosen is that almost all "e-things" will again become simply things as soon as we become acquainted with the novelty of the Internet; after all, we all know that e-commerce is just commerce...

Allen, in a further editorial [5], discovered a new difference: telemedicine remains linked to medical professionals, while e-health is driven by non-professionals, namely patients (or, in the e-health jargon, consumers) that with their interests drive new services even in the healthcare field-mostly for their empowerment through access to information and knowledge .

Interestingly enough, even after the name change, *Telemedicine Journal and eHealth* did not publish any paper directly mentioning e-health; also, the other major scientific journal related to telemedicine, the *Journal of Telemedicine and Telecare*, seems not to care much about e-health. This could be related to the business role the term e-health seems to have: when researchers describe their work, the classical categories of, for example, Medical Informatics, Telemedicine, and Electronic Patient Records are more meaningful than the generic term e-health.

As a researcher, I can see some sense in the term e-health; coming from the integration perspective it suggests: integrated-healthcare-systems' properties, possibilities, and consequences that are (in a holistic approach) more than the sum of the single-component outcomes. However, even these aspects are already studied in some computer science fields - for example Artificial Intelligence (at least inside the multi-agent paradigm), Information Economics, and Dynamic Systems; thus, there is nothing new again, except for the specific interest in healthcare.

*Vincenzo Della Mea*

*Institute of Pathology, University of Udine, Italy*

## References

[1] Mitchell J (2000). Increasing the cost-effectiveness of telemedicine by embracing e-health. J Telemed Telecare.

[2] Mitchell J (1999). From telehealth to e-health: the unstoppable rise of e-health.

[3] Allen A (1999). Editor's note: when the ship.com comes in. Telemed Today.

[4] Rosen E (2000). The death of telemedicine?. Telemed Today.

[5] Allen A (2000). Morphing Telemedicine - Telecare - Telehealth - eHealth. Telemed Today, Special issue: 2000 Buyer's Guide and Directory.

